# Coexisting Sacrococcygeal Teratoma With Mild Encephalitis/Encephalopathy With a Reversible Splenial Lesion: A Case Report

**DOI:** 10.7759/cureus.62574

**Published:** 2024-06-17

**Authors:** Goshi Fujimoto, Takashi Deguchi, Junya Shirai, Kentaro Saito

**Affiliations:** 1 Gastroenterological Surgery, Koga Community Hospital, Yaizu, JPN

**Keywords:** cystic lesions, ciliated columnar epithelium, keratides, teratoma, sacrococcygeal teratoma, splenium, corpus callosum, reversible splenial lesion, mers, encephalitis

## Abstract

Mild encephalitis/encephalopathy with a reversible splenial lesion (MERS) is a rare disease characterized by a reversible lesion in the splenium of the corpus callosum (SCC) observed on MRI. The exact etiology of MERS is unknown, although infections and antiepileptic drugs have been reported as potential causes. Herein, we present the case of a 56-year-old male patient who experienced fever and headache for 3 days. He was referred to our hospital after symptomatic treatment by his primary care physician failed to improve his symptoms. The patient had no psychiatric symptoms or significant neurological findings. Head MRI revealed a high signal on SCC on diffusion-weighted imaging, raising the suspicion of MERS. All examinations to determine the cause of MERS were negative. The patient's symptoms improved with antibiotics and B complex vitamins.

Upon admission, abdominal CT incidentally revealed a well-defined mass on the dorsal surface of the rectum suspected to be a tailgut cyst, warranting surgical resection. The cranial margin of the tumor was caudal to the third sacrum, and a trans-sacral approach was used for resection. The fifth sacrum and the coccyx were resected, and the tumor was resected without damaging the rectum. A histopathological examination revealed a mature teratoma without any malignancy. A follow-up CT at four months postoperatively showed no evidence of clinical recurrence of MERS. Adult-onset MERS is relatively rare, and no association with tumors has been reported. The association between encephalitis and teratomas includes ovarian teratomas, which cause anti-N-methyl-D-aspartate receptor encephalitis and paraneoplastic limbic encephalitis. Although the cause of MERS was unknown in this case, we report the coexistence of a sacral teratoma and MERS to contribute to the knowledge of the association between them.

## Introduction

Mild encephalitis/encephalopathy with a reversible splenial lesion (MERS) is characterized by MRI findings of a reversible lesion in the splenium of the corpus callosum (SCC), sometimes involving symmetrical white matter. Patients with MERS usually exhibit mild central nervous system symptoms such as disturbance of consciousness, seizures, and headache, which recover within a month [1,2]. The exact etiology of MERS is unknown, although infections and antiepileptic drugs have been implicated, with no known association with tumors.

Adult sacrococcygeal tumors are diverse, including benign tumors like epidermoid cysts (33.6%) and mature teratomas (25.5%), as well as malignant tumors such as teratomas with malignant transformation (2.7%) and fibromyxoid sarcoma (1.8%) [3]. Preoperative diagnosis is difficult due to the risk of seeding from biopsy. Therefore, a complete tumor resection is recommended, with the surgical approach determined by the tumor's location [4].

Regarding the association between encephalitis and teratomas, ovarian teratomas cause anti-N-methyl-D-aspartate receptor (NMDAR) encephalitis and paraneoplastic limbic encephalitis (PLE). However, reports on the association between MERS and neoplastic lesions are lacking. We present a case of an adult male with both MERS and a sacrococcygeal mature teratoma to contribute to the understanding of any potential association between these conditions.

## Case presentation

Here we present the case of a 56-year-old male patient who experienced fever and headache. He was referred to our hospital after symptomatic treatment failed to improve his symptoms. His medical history included hypertension and depression, for which he was treated with candesartan cilexetil and amlodipine besylate, trazodone hydrochloride, vortioxetine hydrobromide, lemborexant, and zolpidem tartrate. He had no history of antiepileptic drug use or alcohol consumption, had a Brinkman index of 400 from smoking 16 years ago, and had a body mass index of 27.2 kg/m2. He exhibited no psychiatric symptoms or significant neurological findings. A blood test revealed elevated C-reactive protein (CRP) levels (Table 1).

**Table 1:** Blood test results WBC, white blood cell; RBC, red blood cell; Hb, hemoglobin; Ht, hematocrit; PLT, platelet; PT, prothrombin time; INR, international normalized ratio; APTT, activated partial thromboplastin time; TP, total protein; Alb, albumin; T-Bil, total bilirubin; BUN, blood urea nitrogen; Cre, creatine; LDH, lactate dehydrogenase; CK, creatinine kinase; AST, aspartate aminotransferase; ALT, alanine aminotransferase; γGTP, gamma-glutamyl transpeptidase; AMY, amylase; Na, sodium; K, potassium; Cl, chlorine; CRP, C-reactive protein; CA19-9, carbohydrate antigen 19-9

Laboratory parameter	Result	Reference range
WBCs (/μL)	5670	3300–9000
RBCs (×10^4^/μL)	510	430–570
Hb (g/dL)	14.7	13.5–17.5
Ht (%)	43.8	39.7–52.4
PLTs (×10^4^/μL)	13.8	14.0–34.0
PT (INR)	0.99	0.85–1.15
PT (%)	102.9	70.0–100.0
APTT (s)	33	25.0–36.0
TP (g/dL)	6.9	6.7–8.3
Alb (g/dL)	3.8	3.8–5.2
T–Bil (mg/dL)	1.8	0.2–1.2
BUN (mg/dL)	15.1	8.0–20.0
Cre (mg/dL)	0.8	0.61–1.04
LDH (IU/L)	190	124–222
CK (IU/dL)	57	60–270
AST (IU/L)	29	10–40
ALT (IU/L)	91	5–45
γGTP (IU/L)	347	<80
AMY (IU/L)	37	40–122
Na (mEq/L)	139	137–147
K (mEq/L)	3.8	3.5–5.0
Cl (mEq/L)	103	98–108
CRP (mg/dL)	10.4	<0.30
CA19-9 (U/mL)	37.4	<37.0

**Figure 1:**
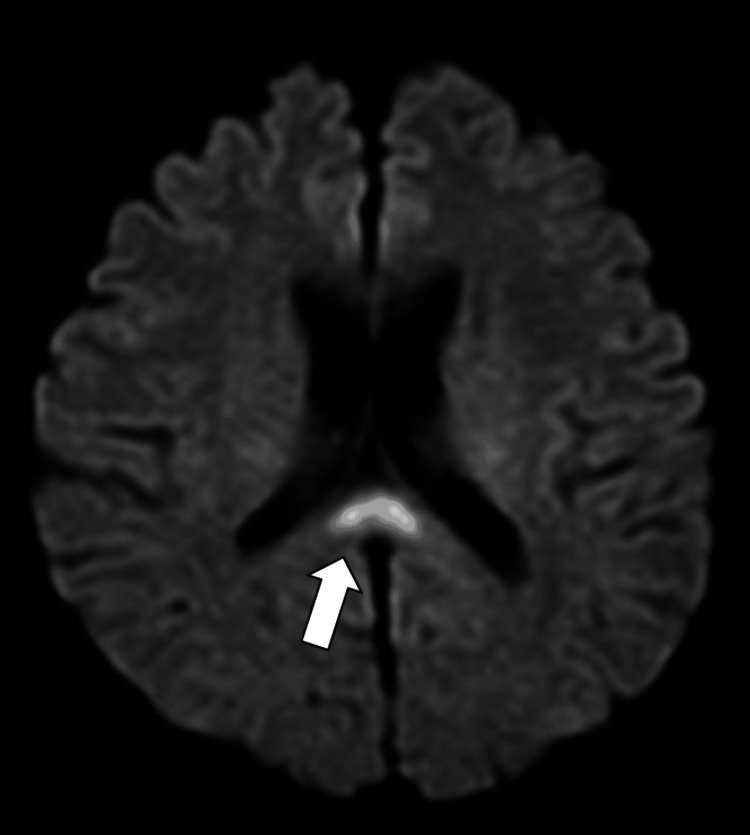
Diffusion-weighted imaging of the head MRI A high signal in the center of the splenium of the corpus callosum (SCC) extending irregularly into the lateral portion of the SCC was revealed (arrow).

Diffusion-weighted imaging (DWI) of the head MRI showed a high signal in the center and lateral portion of the SCC (Figure 1).

Cerebrospinal fluid (CSF) examination showed normal cell count, sugar, and protein levels (Table 2).

**Table 2:** Cerebrospinal fluid test results

Laboratory parameter	Result	Reference range
Cell (/μL)	1	0–5
Protein (mg/dL)	28.3	10–40
Sugar (mg/dL)	63	50–75

Antibiotics (ampicillin-sulbactam) and B complex vitamins (B1, B6, and B12) were administered, and the patient's symptoms and CRP level improved after two days. As no new neurological symptoms were observed, steroid therapy was not administered. The patient underwent blood and spinal fluid tests to search for the cause of the SCC lesion, including human herpesvirus 6, herpes simplex virus (HSV) 1, HSV 2, varicella-zoster virus, and cytomegalovirus as prior infectious diseases; Hashimoto's thyroiditis (anti-thyroglobulin, myeloperoxidase, and thyroid stimulating hormone receptor antibody) and systemic lupus erythematosus (anti-antinuclear antibody, anti-SSA and SSB antibody) as autoimmune diseases; and multiple sclerosis (myelin basic protein) and neuromyelitis optica spectrum disorders (anti-aquaporin 4 antibody) as demyelinating diseases. All tests were negative. Since the testing for anti-NMDAR antibodies is expensive and not covered by insurance, the patient did not undergo the test. Electroencephalography (EEG) showed no significant findings. A follow-up MRI four days after treatment revealed the disappearance of the high-signal lesion on DWI. Based on the clinical course and imaging findings, the patient was diagnosed with MERS.

**Figure 2:**
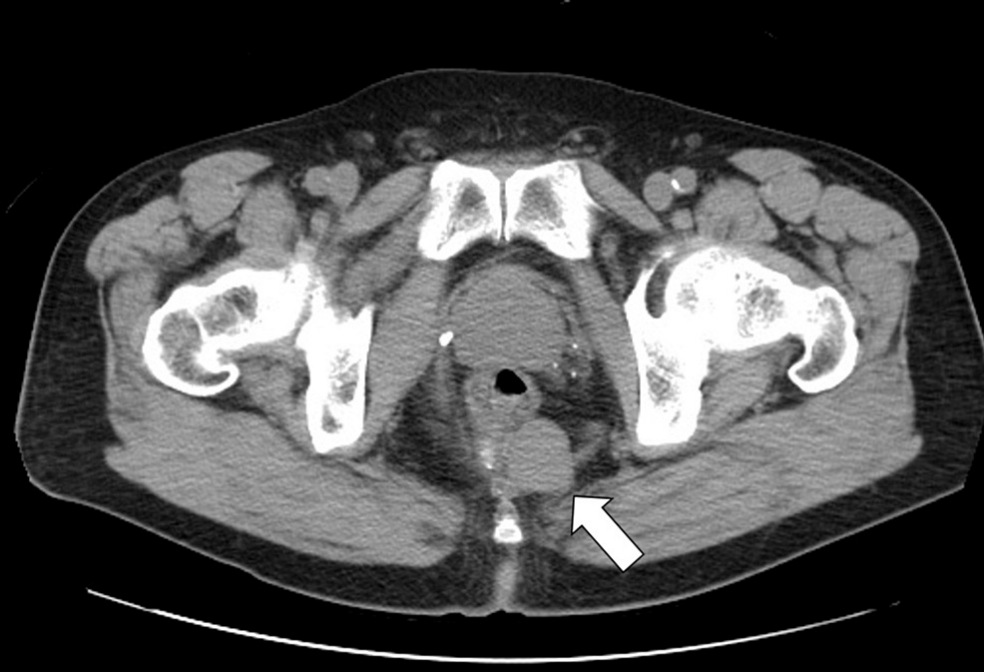
Abdominal CT findings CT incidentally revealed a well-defined multilocular mass (3 cm in diameter) on the dorsal surface of the rectum (arrow).

On admission, an abdominal CT scan, which was performed in the emergency department to determine the cause of the fever, incidentally revealed a well-defined multilocular mass 3 cm in diameter on the dorsal surface of the rectum, and the patient was referred to our department (Figure 2).

**Figure 3:**
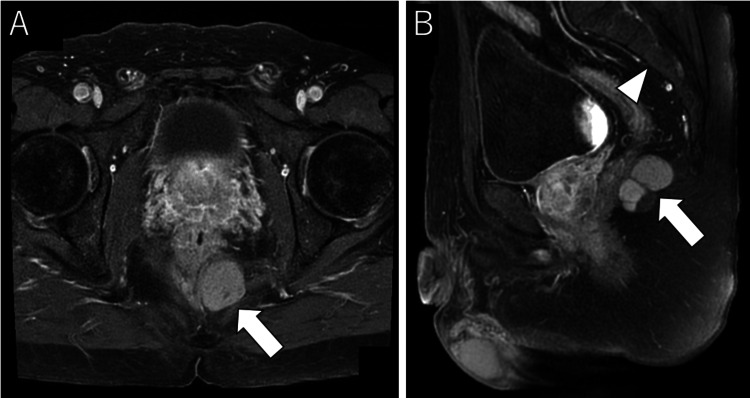
T1-weighted images of abdominal MRI (A) Axial magnetic resonance imaging (MRI) showed a high signal inside the tumor, which had an unclear contrast effect (arrow). (B) Coronal MRI revealed that the tumor (arrow) was caudal to the third sacrum (arrowhead).

Pelvic MRI identified a 3 cm diameter nodular lesion extending from the dorsal rectum to the subcutaneous region of the buttock (Figure 3).

**Figure 4:**
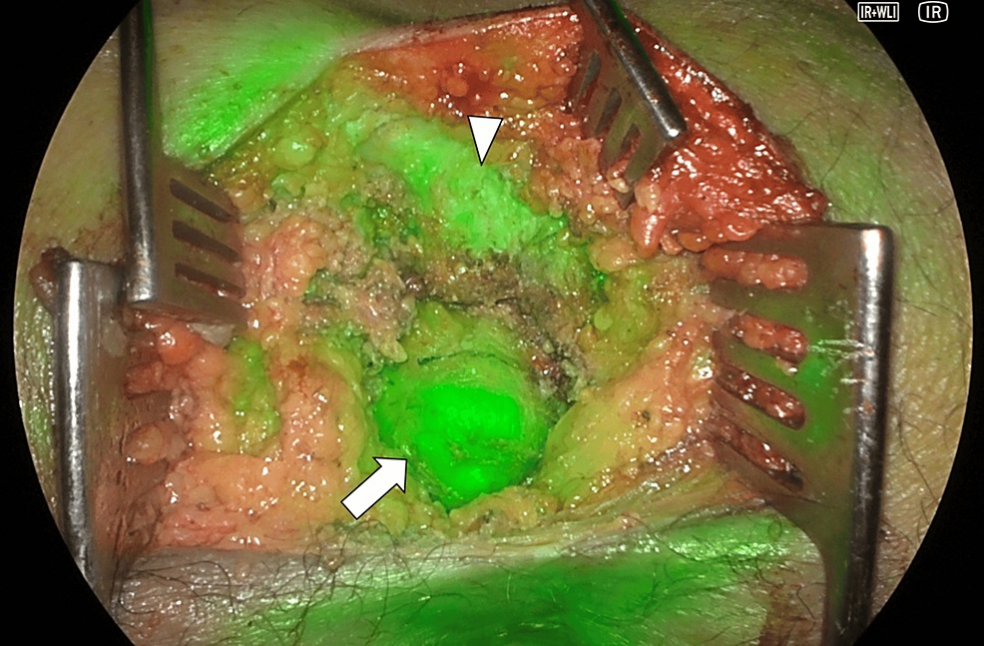
Indocyanine green (ICG) fluorescence imaging ICG imaging showed that blood flow in the rectum (arrow) was maintained. The arrowhead showed a resected margin of the sacrum.

T1-weighted images (T1WI) on MRI showed a high signal inside the tumor with an unclear contrast effect and no signs of malignancy. The preoperative diagnosis was a tailgut cyst, and surgical resection was performed. The cranial margin of the tumor was caudal to the third sacrum; therefore, a trans-sacral approach was employed for resection. A skin incision was made from the left side of the third sacrum to the dorsal anus, and the surgical field was developed with a combined resection of the fifth sacrum and coccyx. The tumor was resected without damaging the rectum, and intraoperative indocyanine green fluorescence imaging demonstrated that blood flow to the rectum was maintained (Figure 4). The operative time was one hour and nine minutes, and the blood loss was 10 mL.

**Figure 5:**
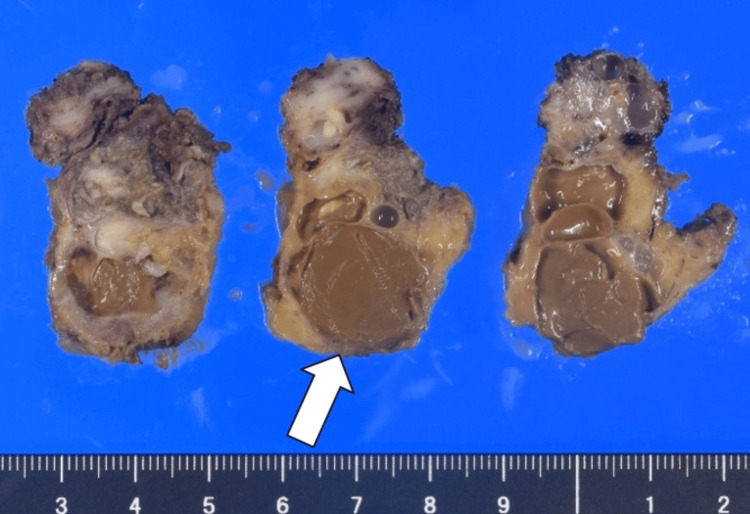
Macroscopic findings The excised specimens were multifocal cystic lesions with brown contents (arrow).

A macroscopic examination of the excised specimen revealed multifocal cystic lesions with brown contents (Figure 5).

**Figure 6:**
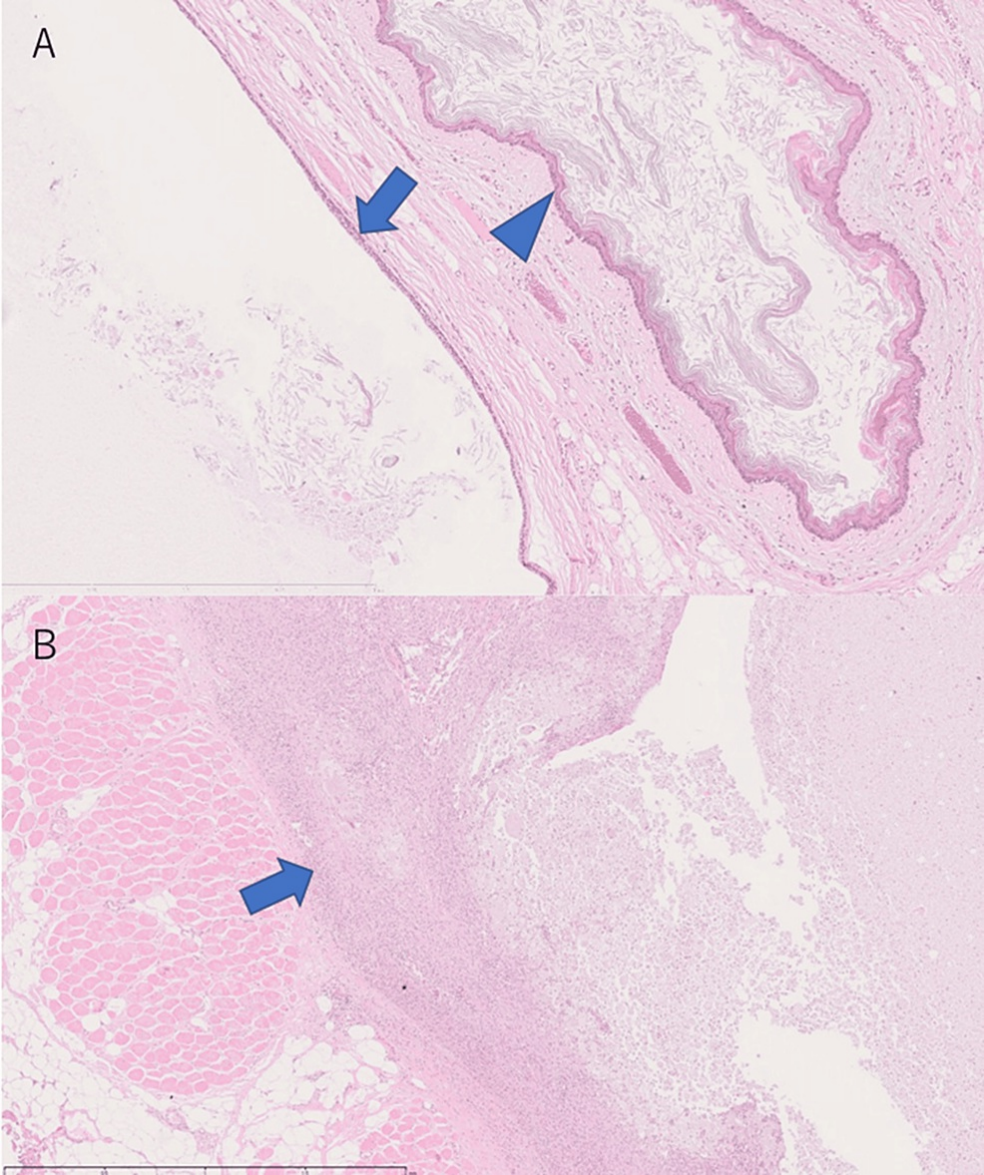
Histopathological findings on H&E-stained sections (A) Microscopic findings revealed cysts lined with stratified squamous epithelium (arrowhead) and cysts lined with ciliated columnar epithelium (arrow), containing stratified keratides and mucus, respectively (H&E stain, original magnification ×40). (B) Some cysts had a foreign body reaction to the keratides around the cysts (arrow) (H&E stain, original magnification ×25)

Histopathological findings showed cysts lined with stratified squamous epithelium and cysts lined with ciliated columnar epithelium, containing stratified keratides and mucus, respectively (Figure 6A). Some cysts showed a foreign body reaction to the keratides around the cysts (Figure 6B).

The lesion originated from a deep midline location and was diagnosed as a mature teratoma. No evidence of immature or malignant features was found.

Follow-up CT at four months postoperatively showed no evidence of tumor recurrence or clinical recurrence of MERS.

## Discussion

Generally, patients with MERS present with mild central nervous system symptoms such as consciousness disturbances, seizures, and headache, with complete recovery usually within a month and about half recovering within a week [1]. In this case, symptoms resolved five days after the onset of fever and four days after the onset of headache, consistent with previous reports. MERS can be divided into two types based on lesion location. The typical form, MERS type I, is often associated with specific lesions in the midline of the SCC, whereas MERS type II often presents with lesions with similar signal symptoms in the symmetrical cerebral white matter or the anterior aspect of the corpus callosum. Typical MRI features include transient high signal intensity on T2WI, fluid-weighted inversion recovery (FLAIR), DWI, decreased apparent diffusion coefficient (ADC) values on ADC maps, and hyper-isointense signals on T1WI sequences without contrast enhancement [1]. In this case, the high signal on DWI extended into the lateral portion of the SCC, classifying it as MERS type II. MERS is caused by influenza virus, rotavirus, O-157 E. coli [5], MRSA endocarditis [6], Legionella [7], mumps virus, Mycoplasma pneumoniae, ticks, and antiepileptic drugs [1]. Other possible causes include high-altitude cerebral edema, cesarean section, and hyponatremia [2]. However, the exact pathogenesis of MERS remains unknown. Several hypotheses have been proposed, including intramyelinic edema, axonal damage, hyponatremia, and oxidative stress. A possible explanation for these MRI findings is intramyelinic edema resulting from the separation of myelin layers and local infiltration of inflammatory cells [1].

Other differential diagnoses include posterior reversible encephalopathy syndrome (usually associated with hypertension and subcortical white matter lesions), multiple sclerosis (with a characteristic relapsing-remitting course), Marchiafava-Bignami disease (often seen in alcoholics), ischemia (usually irreversible with vascular territory distribution), diffuse axonal injury (associated with head trauma), lymphoma (contrast-positive), and extracerebral spinal cord dissection (with electrolyte abnormalities) [1,6]. The patient had no significant findings on blood tests, infectious disease tests, autoimmune disease markers, demyelinating disease markers, CSF tests, or EEG, and had no history of alcohol consumption. Based on the clinical and reversible MRI findings, the patient was diagnosed with MERS, although the exact cause remained unknown.

Methylprednisolone and intravenous immune globulin (IVIG) are common treatments for MERS; however, patients who do not use either methylprednisolone or IVIG are also clinically cured, and the efficacy of these treatments remains uncertain [1,2].

The sacrococcygeal tumor noted incidentally in this case was suspected to be a tailgut cyst based on the CT and MRI findings. Biopsy of tailgut cysts carries the risks of seeding, infection, and intraperitoneal dissemination. Although the rate is very low (< 5%), it can become malignant [8]. Imaging features suggestive of a concomitant infection or malignancy include nodular wall thickening and enhancement, intracystic vegetation, indistinct margins, cranial extension above the S3 level, and associated lymphadenopathy, none of which were present in this case [9]. For cysts that can be completely resected, a preoperative biopsy should not be performed; they should be resected en bloc as a “large biopsy.” [10]

Histopathologic examination of this case confirmed a diagnosis of mature teratoma, not diagnosed preoperatively. Primary retroperitoneal teratomas account for 1-11% of all retroperitoneal neoplasms [11]. Most teratomas are benign, and approximately 1-2% undergo malignant transformation, including squamous cell carcinoma, adenocarcinoma, sarcoma, and other malignancies [12]. CT and MRI are effective for detecting presacral space-occupying lesions and assessing their relationship to the rectum and sacrum. MRI can differentiate between benign and malignant tumors with specificity and sensitivity of 97% and 88%, respectively [12]. Teratoma treatment generally involves surgical resection upon detection. Teratomas are classified as mature or immature according to the degree of differentiation of their components, with immature tumors being more likely to exhibit malignant behavior in adults [13].

The surgical approaches for sacrococcygeal tumors include the sacrococcygeal approach, single abdominal approach, combined sacrococcygeal approach, and anal approach, selected based on the tumor's anatomical location. In this case, the tumor was localized caudal to S3; therefore, a sacrococcygeal approach combined with coccygeal resection was used to secure the operative field. Conversely, the single abdominal approach is useful for tumors cranial to S3, whereas the combined sacrococcygeal approach, including the posterior sacral opening and abdominal anatomy, is effective for larger resections in restricted regions such as the posterior rectal space [4].

In this case, an adult male presented with a coexisting mature sacrococcygeal teratoma and MERS, and whether the mature teratoma directly caused MERS remains unclear. Associations between teratoma and encephalitis have been reported, such as ovarian teratomas with NMDAR encephalitis and PLE [14,15].

Patients with encephalitis associated with antibodies against NMDARs are usually young adults, primarily women and children, with rapidly progressive symptoms of psychosis, abnormal movements, autonomic dysfunction, and coma. Anti-NMDAR encephalitis is characterized by the presence of antibodies against the GluN1 subunit of the NMDAR, although the probability of antibody detection is less than 50% in patients over age 50 [16,17]. An abnormal brain MRI was defined as acute cortical, frontal, striatal, or temporal T2WI/FLAIR hyperintensities, which were absent in 51% of patients with NMDAR encephalitis [18]. In this case, since the MRI findings did not indicate anti-NMDAR encephalitis and the testing for anti-NMDAR antibodies is a significant economic burden on the patient, the patient did not undergo the test. Mature ovarian teratomas and herpes simplex encephalitis primarily trigger NMDAR autoimmunity [14,18]. The coexistence of NMDAR encephalitis and tumors in women includes mature or immature ovarian teratomas, sex-cord stromal tumors, neuroendocrine tumors, and mediastinal teratomas. In men, it includes small-cell lung cancer, immature teratoma of the testis, prostate cancer, and CNS lymphoma [18].

PLE is a rare disorder characterized by personality changes, irritability, depression, seizures, memory loss, and dementia. Diagnosing PLE requires either a neuropathological examination or meeting the following four criteria: (i) a compatible clinical picture; (ii) an interval of less than four years between the onset of neurologic symptoms and tumor diagnosis; (iii) exclusion of other neuro-oncologic complications; and (iv) at least one of the following: CSF with inflammatory changes and negative cytology, MRI showing abnormalities in the temporal lobe, and EEG showing epileptic activity in the temporal lobe. Commonly associated neoplasm sites include the lungs (50%), testes (20%), and breasts (8%), although cases of coexisting immature ovarian teratomas have also been reported [15].

Although anti-NMDAR encephalitis, PLE, and MERS differ in their typical imaging findings and clinical courses, teratomas may have triggered MERS. The histopathological findings in this case, showing a foreign body reaction to keratides around some of the cysts, were similar to inflammatory cell infiltration in an ovarian teratoma that developed NMDAR encephalitis [19]. Further case studies are needed to investigate the association between teratomas and MERS.

## Conclusions

We encountered a case involving the surgical resection of a sacrococcygeal mature teratoma in an adult male patient, which was incidentally revealed during an episode of MERS. The diagnosis of MERS entails confirming typical clinical and imaging findings while excluding numerous differential diagnoses. Preoperative diagnoses of sacrococcygeal tumors are challenging, and the choice of surgical approach depends on tumor location, aiming for complete resection. Although the cause of MERS remained unknown in this case, we presented this instance of teratoma and MERS coexistence to contribute to the understanding of their potential association. Further studies are needed to confirm the association between teratomas and MERS.

## References

[1] Yuan J, Yang S, Wang S, Qin W, Yang L, Hu W (2017). Mild encephalitis/encephalopathy with reversible splenial lesion (MERS) in adults-a case report and literature review. BMC Neurol.

[2] Pan JJ, Zhao YY, Lu C, Hu YH, Yang Y (2015). Mild encephalitis/encephalopathy with a reversible splenial lesion: five cases and a literature review. Neurol Sci.

[3] Zhao X, Zhou S, Liu N, Li P, Chen L (2022). Is there another posterior approach for presacral tumors besides the Kraske procedure? - a study on the feasibility and safety of surgical resection of primary presacral tumors via transsacrococcygeal transverse incision. Front Oncol.

[4] Liang F, Li J, Yu K, Zhang K, Liu T, Li J (2020). Tailgut cysts with malignant transformation: features, diagnosis, and treatment. Med Sci Monit.

[5] Tada H, Takanashi J, Barkovich AJ (2004). Clinically mild encephalitis/encephalopathy with a reversible splenial lesion. Neurology.

[6] Hagiya H, Otsuka F (2022). Adult-onset mild encephalitis/encephalopathy with a reversible Splenial lesion induced by MRSA endocarditis. Eur J Case Rep Intern Med.

[7] Shimono H, Hoshina Y, Ogawa E (2021). A rare etiology of mild encephalitis/encephalopathy with reversible splenial lesion. Clin Case Rep.

[8] Saba L, Fellini F, Greco FG (2014). MRI evaluation of not complicated tailgut cyst: case report. Int J Surg Case Rep.

[9] Shetty AS, Loch R, Yoo N, Mellnick V, Fowler K, Narra V (2015). Imaging of tailgut cysts. Abdom Imaging.

[10] Patsouras D, Pawa N, Osmani H, Phillips RK (2015). Management of tailgut cysts in a tertiary referral centre: a 10-year experience. Colorectal Dis.

[11] Shin NY, Kim MJ, Chung JJ, Chung YE, Choi JY, Park YN (2010). The differential imaging features of fat-containing tumors in the peritoneal cavity and retroperitoneum: the radiologic-pathologic correlation. Korean J Radiol.

[12] Guo JX, Zhao JG, Bao YN (2022). Adult sacrococcygeal teratoma: a review. Medicine (Baltimore).

[13] Peterson CM, Buckley C, Holley S, Menias CO (2012). Teratomas: a multimodality review. Curr Probl Diagn Radiol.

[14] Dalmau J, Armangué T, Planagumà J (2019). An update on anti-NMDA receptor encephalitis for neurologists and psychiatrists: mechanisms and models. Lancet Neurol.

[15] Gultekin SH, Rosenfeld MR, Voltz R, Eichen J, Posner JB, Dalmau J (2000). Paraneoplastic limbic encephalitis: neurological symptoms, immunological findings and tumour association in 50 patients. Brain.

[16] Guasp M, Dalmau J (2018). Encephalitis associated with antibodies against the NMDA receptor. Med Clin (Barc).

[17] Kaneko A, Kaneko J, Tominaga N (2018). Pitfalls in clinical diagnosis of anti-NMDA receptor encephalitis. J Neurol.

[18] Nissen MS, Ørvik MS, Nilsson AC, Ryding M, Lydolph M, Blaabjerg M (2022). NMDA-receptor encephalitis in Denmark from 2009 to 2019: a national cohort study. J Neurol.

[19] Abdul-Rahman ZM, Panegyres PK, Roeck M (2016). Anti-N-methyl-D-aspartate receptor encephalitis with an imaging-invisible ovarian teratoma: a case report. J Med Case Rep.

